# Childhood MMR vaccination and the incidence rate of measles infection: a ten year longitudinal cohort study of American children born in the 1990s

**DOI:** 10.1186/s12887-019-1710-5

**Published:** 2019-09-10

**Authors:** David A. Geier, Janet K. Kern, Mark R. Geier

**Affiliations:** 1 Institute of Chronic Illnesses, Inc, 14 Redgate Ct, Silver Spring, MD 20905 USA; 2 CoMeD, Inc, 14 Redgate Ct, Silver Spring, MD 20905 USA

**Keywords:** Cohort, Measles, MMR vaccine, Pediatric, Rubeola

## Abstract

**Background:**

Measles (rubeola) is a highly contagious disease with significant morbidity/mortality. Measles-Mumps-Rubella (MMR) is a live-attenuated vaccine used in the United States (US) since the early 1970s to prevent measles infection. This retrospective longitudinal cohort study examined childhood MMR vaccination effectiveness (VE) on preventing diagnosed measles cases.

**Methods:**

The Independent Healthcare Research Database (IHRD) is composed of non-identifiable linked eligibility and claim healthcare records prospectively generated from the Florida Medicaid system. The SAS system was utilized to examine a cohort of 101,736 persons eligible for Florida Medicaid from 1990 to 2009 and continuously eligible with ≥10 outpatient office visits during the 120-month period following birth. There were 32,870 persons (224,492 person-years) in the cohort receiving a single dose of childhood MMR vaccine (vaccinated) and 43,538 persons (434,637 person-years) in an unvaccinated cohort (no exposures to measles-containing vaccine). The frequency of diagnosed measles (ICD-9 code: 055xxx) was examined. Cox proportional hazards models evaluated MMR vaccination and diagnosed measles over time.

**Results:**

MMR vaccinated cohort members were at significantly reduced risk of measles in the unadjusted (VE = 83.6, 95% CI = 67.2–91.8%) and adjusted (VE = 80.7, 95% CI = 61.5–83.9%) models as compared to the unvaccinated cohort. VE = 80% among younger MMR recipients (12–15 months), whereas VE = 90% among older MMR recipients (16–20 months) as compared to the unvaccinated cohort.

**Conclusion:**

Routine childhood MMR vaccination significantly reduced the incidence rate of childhood measles infections, and the VE was greater in the older recipients (16–20 months) than in the younger recipients (12–15 months).

## Background

As described by the United States (US) Centers for Disease Control and Prevention (CDC), measles (rubeola) is highly contagious (90% of exposed susceptible persons develop measles) rash illness that is transmitted by direct contact with respiratory droplets or airborne spread between person to person [1]. Measles was observed to occur in epidemic cycles and virtually all people acquired measles before adulthood in the US prior to the implementation of the national measles vaccine program in 1963. It was described prior to the national measles vaccine program in the US, annually about 500,000 cases of measles were reported, of whom 500 persons died, 48,000 were hospitalized, and about 1000 cases of encephalitis with permanent brain damage were observed [2].

In the US, measles vaccination was initially recommended for administration at 9 months in 1963, 12 months in 1965, and 15 months in 1967 [1]. During the 1970s combined measles-mumps-rubella (MMR) vaccine was introduced in the US [3, 4]. The MMR vaccine used in the US since the 1980s is the M-M-R® II vaccine (Merck & Co, Inc., Whitehouse Station, NJ), and is a sterile lyophilized preparation of (1) ATTENUVAX® (Measles Virus Vaccine Live), a more attenuated line of measles virus, derived from Enders’ attenuated Edmonston strain and propagated in chick embryo cell culture; (2) MUMPSVAX® (Mumps Virus Vaccine Live), the Jeryl Lynn™ (B level) strain of mumps virus propagated in chick embryo cell culture; and (3) MERUVAX® II (Rubella Virus Vaccine Live), the Wistar RA 27/3 strain of live attenuated rubella virus propagated in WI-38 human diploid lung fibroblasts [5].

Since, the late 1980s/early 1990s, the Advisory Committee on Immunization Practices (ACIP), the American Academy of Pediatrics (AAP), and American Academy of Family Practitioners (AAFP) have recommended that the first dose of MMR vaccine should be given to children aged 12 through 15 months (with administration between 6 and 12 months under special circumstances) [1].

The measles vaccine program in the US was so successful against measles infections that it is the largest country in the world to have ended endemic measles transmission [6]. Therefore, it would be ethically unacceptable to conduct placebo-controlled trials to assess measles vaccine effectiveness in the US, and as a result, current epidemiological research on the effectiveness of MMR vaccine needs to focus on retrospective studies of populations to optimize protection by modifying immunization schedules [7].

The purpose of the present retrospective longitudinal cohort study was to examine the vaccine effectiveness of childhood MMR vaccination to reduce the incidence rate of childhood measles infections in the US during the 1990s/2000s. In addition, this study examined the relationship between the age of childhood MMR vaccination and its impact on the effectiveness of the vaccine.

## Methods

### Independent healthcare research database (IHRD)

The Independent Healthcare Research Database (IHRD) is composed of non-identifiable healthcare records generated from the Florida Medicaid system. The data contained within the IHRD were obtained from the Agency for Health Care Administration (AHCA) of the state of Florida and included eligibility and claim files. It is possible to link a person’s eligibility and claim records by a unique recipient identifier code. The eligibility records included detailed information for each person regarding their month and year of enrollment, gender, date of birth, and county level residency. The claims records included detailed information for each person regarding their diagnosis status using the International Code for Disease, 9th revision (ICD-9) codes, healthcare procedure codes (medical, dental, etc.), and administered drugs using National Drug Codes (NDC). The data in the IHRD were assembled and accessed under approval by the Liberty Institutional Review Board (IRB) (Deland, FL). The SAS system for Windows, version 9.4 (Cary, NC, USA) was used to examine the IHRD.

#### Study participants

**Fig. 1:**
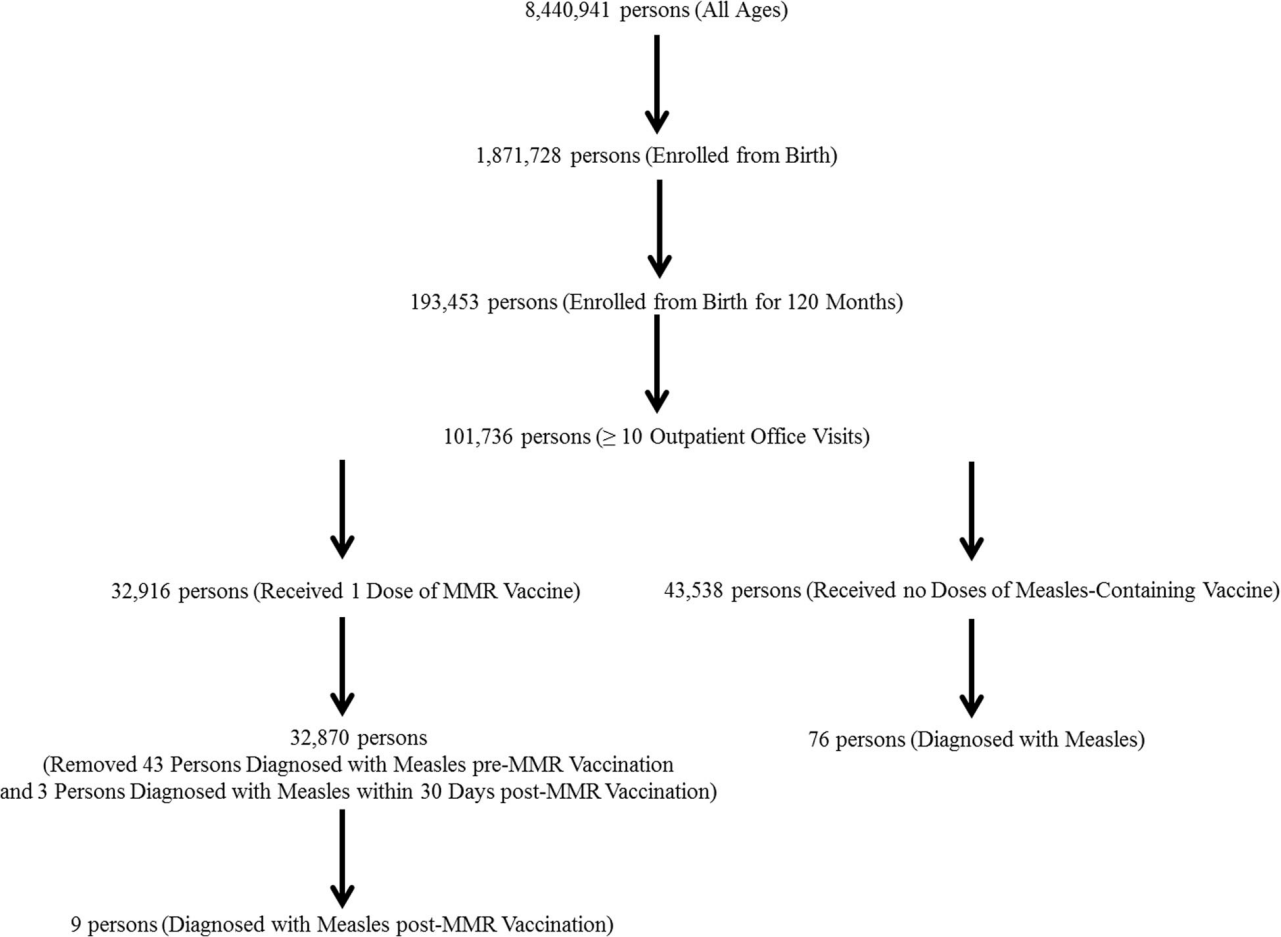
A schematic flowchart of the data examined in the present study. Persons in the MMR vaccinated cohort received only 1 dose of MMR vaccine and includes only persons diagnosed with measles post-MMR vaccine administration (43 persons were excluded with measles diagnoses pre-MMR vaccine administration and 3 persons were diagnosed measles within 30 days of vaccine administration, which are most likely measles vaccine-associated adverse events)

Figure 1 presents a schematic flowchart of the IHRD data examined in the present study. A cohort of 8,440,941 persons of all ages with no changes or missing genders or dates of birth and eligible at specific times for Florida Medicaid from July 1990 through June 2009 was initially evaluated in this study. Among this cohort, a total of 1,871,728 persons were eligible for Florida Medicaid from their date of birth and among those persons a total of 193,453 persons were continuously eligible for Florida Medicaid for 120 months following birth. Finally, among the cohort of 193,453 persons continuously eligible for Florida Medicaid for 120 months following birth, a sub-cohort of 101,736 persons with ≥10 outpatient office visits during the 120-month period following birth was identified.

#### Vaccination status

The exposure variable examined in this study was identified from the healthcare procedure codes filed on claims for each cohort member examined. The procedure codes examined, included: measles vaccination (codes: 9945, W1941, 90705), MMR vaccine (codes: 90707, W1943, 9948), measles and rubella vaccine (code: 90708), measles, mumps, rubella, and varicella (MMRV) vaccine (code: 90710). Only persons receiving a single dose of MMR vaccine were included in the vaccinated cohort (codes: 90707, W1943, or 9948) and persons were considered unvaccinated, if they did not receive any measles-containing vaccine (codes: 9945, W1941, 90705, 90707, W1943, 9948, 90708, or 90710) during the study period examined. All persons receiving more than 1 dose of MMR vaccination or other measles-containing vaccines were excluded from the present study. Among those vaccinated with MMR, the date of service for the first claim in chronological order with a procedure code specifying MMR vaccine was assumed to be the date of vaccine administration. Overall, it was observed that 32,916 persons were in the MMR vaccinated cohort and 43,538 persons were in the unvaccinated cohort.

#### Outcomes

The outcome variable examined in this study was identified from the ICD-9 codes filed on claims for each cohort member examined. All measles-related diagnoses (code: 055xxx) were examined, including: measles (055), post-measles encephalitis (code: 055.0), post-measles pneumonia (code: 055.1), post-measles otitis media (code: 055.2), measles with other specified complications (code: 055.7), measles keratoconjunctivitis (code: 055.71), measles with other specified complications (code: 055.79), measles with unspecified complication (code: 055.8), and measles without mention of complication (code: 055.9). No information was available regarding whether measles cases were laboratory confirmed or not. Persons were considered to have measles if they had any of the measles-related diagnoses and persons were not considered to have measles if they did not have any of the measles-related diagnoses during the study period examined. Among those with a measles diagnosis, the date of service for the first claim in chronological order with a measles diagnosis was assumed to be the date of onset of measles infection. Only those persons either not diagnosed with measles or diagnosed with measles more than 30 days post-MMR vaccination were included in the final MMR vaccinated cohort. As a result, the overall size of the MMR vaccinated cohort was reduced to 32,870 persons.

#### Statistical analyses

In all statistical analyses, the statistical package in SAS was utilized, and a two-sided *p*-value < 0.05 was considered statistically significant. The null hypothesis was that MMR vaccination would have no impact on the incidence rate of measles diagnoses. It was also assumed in this study that chances of exposure to wild type measles virus were equal in the vaccinated and unvaccinated cohorts.

In order to evaluate the relationship between MMR vaccination and diagnosed measles, person-years of follow-up were calculated in the vaccinated and unvaccinated cohorts examined in this study. In the unvaccinated cohort, person-years of follow-up began on the date of birth and continued until the end of eligibility (a maximum of 120 months after birth) or until the date of the first measles diagnosis. In the vaccinated cohort, person-years of follow-up began on the date of MMR vaccine administration and continued until the end of eligibility (a maximum of 120 months after birth) or until the date of the first measles diagnosis.

A regression analysis of diagnosed measles cases based on the Cox proportional hazards model was used to evaluate overtime in years the potential relationship between MMR vaccination and the outcome of a measles diagnosis. Ties in the failure times were handled using the exact method. In addition, an evaluation of the potential impact of young (< 1 year-old) persons in the unvaccinated cohort being diagnosed with measles at an age prior to the earliest age when MMR vaccine is first recommended at 12 months-old was undertaken. MMR vaccine effectiveness was examined in modeling by only counting cases of measles diagnosed at ≥12 months-old in the unvaccinated cohort and only examining persons receiving MMR vaccine at ≥12 months-old in the vaccinated cohort. Finally, modeling was conducted to evaluate the potential impact of age of MMR vaccine administration (≥ 12 months, ≥ 16 months, and ≥ 20 months) on the effectiveness of the vaccine to prevent cases of measles. All models were constructed without adjustment for covariates (Model I) and with adjustment for the covariates of gender (categorical variable), date of birth (continuous variable), and county of residence (as a continuous variable) (Model II). Overall, vaccine effectiveness was determined as ((1 – hazard ratio) × 100).

## Results

Table 1 displays the demographic characteristics of the population of persons examined in this study. Overall, there were a total 32,870 persons in the MMR vaccinated cohort contributing a total of 224,492 person-years and 43,538 persons in the unvaccinated cohort contributing a total of 434,637 person-years. The gender distribution was similar in the MMR vaccinated cohort (male/female ratio: 1.13) and unvaccinated cohort (male/female ratio = 1.14). In addition, overall mean dates of birth were similar in both the vaccinated and unvaccinated cohorts.

**Table 1:** Demographic characteristics of the persons examined in this study^a^

Parameter Examined	MMR Vaccinated Cohort^b^ (*n* = 32,870)	Unvaccinated Cohort^c^ (*n* = 43,538)
Person-Years	224,492	434,637
Gender (%)		
Male	17,468 (53.14%)	23,202 (53.29%)
Female	15,402 (46.86%)	20,336 (46.71%)
Date of Birth		
mean ± std. (range)	1995 ± 2.5 (1990–1999)	1994 ± 2.5 (1990–1999)
Number Diagnosed with Measles (ICD-9 Code: 055xxx)	9	76

*ICD-9* International Code of Disease, 9th revision, *MMR* Measles, mumps, rubella, *std*. Standard deviation

^a^ All persons examined in this study were enrolled from their date of birth for 120 consecutive months. All persons had non-changing dates of birth and gender status. All persons had ≥10 outpatient office visits

^b^ Persons received only 1 dose of MMR vaccine and includes only persons diagnosed with measles post-MMR vaccine administration (43 persons were excluded with measles diagnoses pre-MMR vaccine administration and 3 persons were diagnosed measles within 30 days of vaccine administration, which are most likely measles vaccine-associated adverse events)

^c^ Persons received no doses of any measles-containing vaccine

Table 2 shows the demographic characteristics of the 85 persons diagnosed with measles examined this study. Slightly more males than females were diagnosed with measles (male/female ratio = 1.15), but the ratio was consistent with those observed in the vaccinated and unvaccinated cohorts examined. The mean date of birth among persons diagnosed with measles was slightly earlier in chronological time in the unvaccinated cohort (1993) as compared to vaccinated cohort (1994). Most diagnosed cases of measles (> 90%) were without complications.

**Table 2:** Demographic summary of the persons diagnosed with measles examined in this study^a^

Parameter Examined	All Persons Diagnosed with Measles (*n* = 85)	Vaccinated Persons Diagnosed with Measles (*n* = 9)^c^	Unvaccinated Persons with Diagnosed Measles (*n* = 76)^d^
Gender (%)			
Male	45 (53.41%)	5 (55.56%)	40 (52.63%)
Female	40 (47.06%)	4 (44.44%)	36 (47.37%)
Date of Birth			
mean ± std. (range)	1993 ± 2.1 (1990–1999)	1994 ± 1.26 (1992–1996)	1993 ± 2.2 (1990–1999)
Age at Measles Diagnosis			
mean ± std. (range)	1.65 ± 1.69 (0.15–7.08)	1.27 ± 1.72 (0.15–5.39)	1.69 ± 1.7 (0.18–7.08)
Year of Measles Diagnosis			
mean ± std. (range)	1995 ± 2.78 (1991–2003)	1996 ± 1.9 (1993–1999)	1994 ± 2.8 (1991–2003)
Measles Diagnosis-Associated Complications			
No Complications	78 (91.77%)	8 (88.89%)	70 (92.11%)
Complications Specified^b^	5 (5.88%)	1 (11.11%)	4 (5.26%)
Unknown Complication Status	2 (2.35%)	0 (0%)	2 (2.63%)

^a^All persons examined in this study were enrolled from their date of birth for 120 consecutive months. All persons had non-changing dates of birth and gender status. All persons had ≥10 outpatient office visits

^b^This includes persons with post-measles otitis media (*n* = 2), measles keratoconjunctivitis (*n* = 1), measles with other specified complications (*n* = 1), measles with unspecified complication (*n* = 1)

^c^Persons received only 1 dose of MMR vaccine and includes only persons diagnosed with measles post-MMR vaccine administration (43 persons were excluded with measles diagnoses pre-MMR vaccine administration and 3 persons were diagnosed measles within 30 days of vaccine administration, which are most likely measles vaccine-associated adverse events)

^d^Persons received no doses of measles-containing vaccine

Table 3 reveals the Cox proportional hazards model results examining the impact of childhood MMR vaccination on the incidence rate of diagnosed measles. It was observed regardless of the age of childhood MMR vaccination that the vaccine effectiveness was 83.6% (95% confidence interval = 67.2 to 91.8%) in the unadjusted and 80.7% (95% confidence interval = 61.5 to 90.4%) in the adjusted models. MMR vaccine effectiveness remained significant when only counting cases of measles diagnosed at ≥1 year-old in the unvaccinated cohort and only examining persons receiving MMR vaccine at ≥1 year-old in the vaccinated cohort in the unadjusted (vaccine effectiveness = 70.9, 95% confidence interval = 37.8 to 86.4%) and adjusted (vaccine effectiveness = 65.4, 95% confidence interval = 25.6 to 83.9%) models.

**Table 3:** Cox proportional hazards model results examining the relationship between MMR vaccination and diagnosed measles

Model	Variable	Hazard Ratio (95% CI)	VE (95% CI)	*p*-value	χ^2^
I	*Vaccinated*^*a*^ vs *Unvaccinated*^*b*^ *(all ages)*	*0.164* *(0.082 to 0.328)*	*83.6%* *(67.2 to 91.8%)*	*< 0.0001*	*26.2*
	*Vaccinated* vs *Unvaccinated* *(≥ 12 months-old)* ^*c*^	*0.291* *(0.136 to 0.622)*	*70.9%* *(37.8 to 86.4%)*	*0.0015*	*10.1*
II	*Vaccinated* vs *Unvaccinated (all ages)*	*0.193* *(0.096 to 0.385)*	*80.7%* *(61.5% to 90.4)*	*< 0.0001*	*21.7*
	Gender (Female vs Male)	1.01 (0.659 to 1.544)		0.97	0.002
	County of Residence	0.993 (0.982 to 1.004)		0.24	1.40
	*Date of Birth*	*0.999* *(0.999 to 0.999)*		*< 0.0001*	*30.2*
	*Vaccinated* vs *Unvaccinated* *(≥ 12 months-old)* ^*c*^	*0.346* *(0.161 to 0.744)*	*65.4%* *(25.6 to 83.9%)*	*0.00065*	*7.39*
	Gender (Female vs Male)	0.959 (0.544 to 1.692)		0.89	0.02
	County of Residence	0.997 (0.982 to 1.011)		0.65	0.21
	*Date of Birth*	*0.999* *(0.999 to 0.999)*		*< 0.0001*	*18.8*

*Italicized* results are statistically significant. Model I = unadjusted, Model II = adjusted for gender, county of residence, and date of birth

*CI* Confidence interval, *VE* Vaccine effectiveness

^a^Persons received only 1 dose of MMR vaccine

^b^Persons received no doses of measles-containing vaccine

^c^Only persons diagnosed with measles at ≥12 months-old were included in the unvaccinated cohort and only examining persons receiving MMR vaccine at ≥12 months-old in the vaccinated cohort, so as to ensure direct overlap in ages with the vaccinated and unvaccinated cohorts

**Fig. 2:**
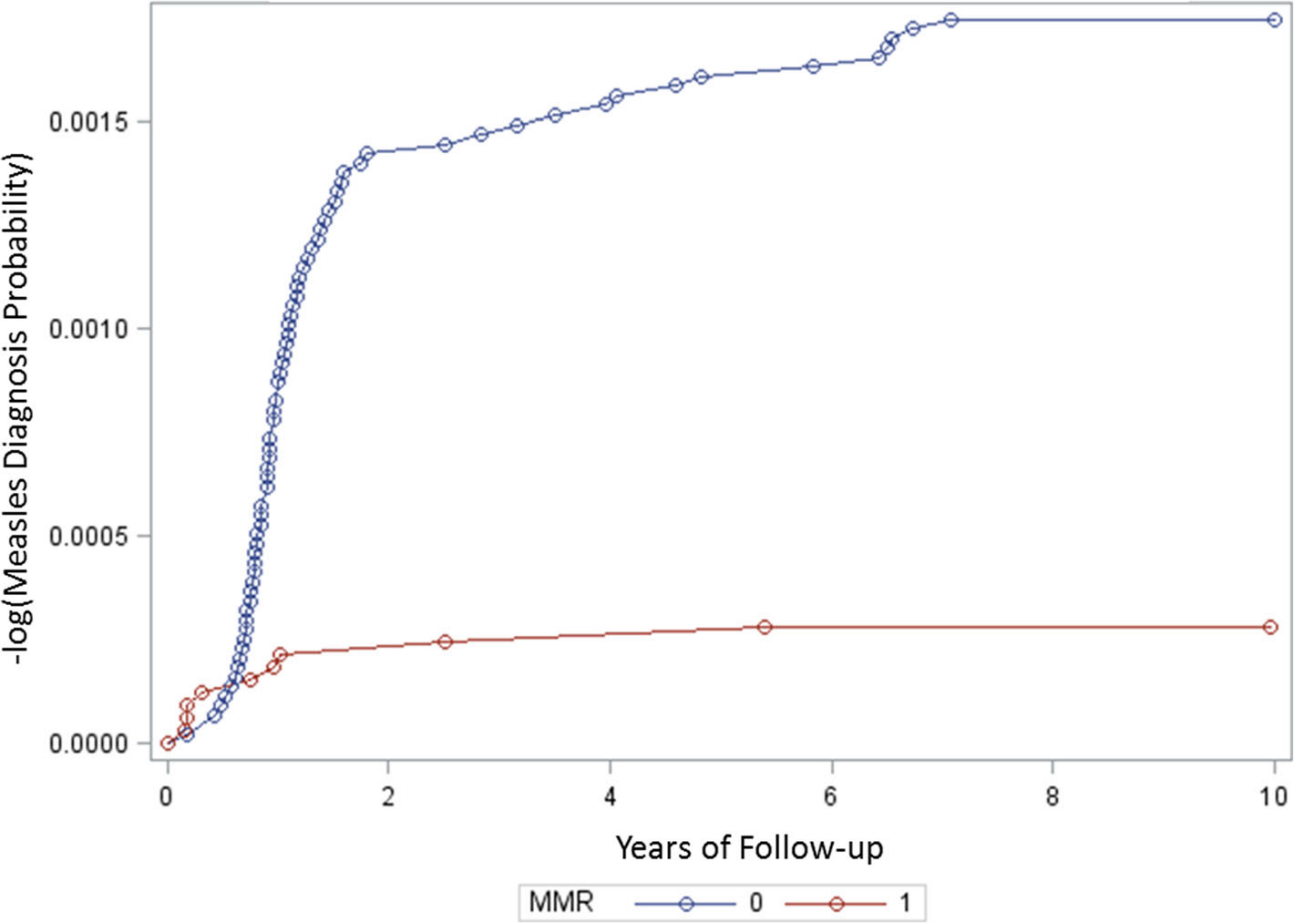
A Cox proportional hazards survival plot evaluating cases of measles diagnosed over the period of follow-up in the MMR vaccinated cohort^1^ (1) in comparison to the unvaccinated cohort^2^ (0). ^1^ Persons received only 1 dose of MMR vaccine. ^2^ Persons received no doses of measles-containing vaccine

Figure 2 is a Cox proportional hazards survival plot evaluating the incidence of measles cases diagnosed over the period of years persons were followed in the MMR vaccinated cohort and the unvaccinated cohort. The plot reveals that in the initial period post-MMR vaccination (< 6 months) there were a greater number of measles cases diagnosed than in the unvaccinated cohort, but in the later periods post-MMR vaccination (> 6 months) there relatively few measles cases diagnosed as compared to the initial 6-month follow-up period in the MMR vaccinated cohort or the unvaccinated cohort. The unvaccinated cohort revealed the most significant increases in the number of measles cases diagnosed in the first 2 years of follow-up in the present study. This was then followed by a period of still increasing numbers of measles case diagnoses from the 2nd to the 6th year of follow-up, but at a slower rate than the initial 2-year period of follow-up. Finally, in the last period from the 6th to 10th year of follow-up, there were relatively few measles cases diagnosed as compared to the previous follow-up periods.

Table 4 reveals the impact of the age of MMR vaccine administration on vaccine effectiveness in comparison to the unvaccinated cohort. Among those persons receiving MMR vaccine at the younger ages examined with receipt at ≥12 months-old was the least effective with a vaccine effectiveness ranging from about 80 to 85%. By contrast, when examining the MMR vaccinated cohort at the older ages examined with receipt at ≥16 months-old or ≥ 20 months-old vaccine effectiveness was about 90% or more.

**Table 4:** An evaluation of the impact of the age of MMR vaccine administration on the effectiveness of the vaccine to prevent cases of measles

Model	Variable	Hazard Ratio (95% CI)	VE (95% CI)	*p*-value	χ^2^
I	*Vaccinated*^*a*^ vs *Unvaccinated*^*b*^ *(≥ 12 months-old)*	*0.147* *(0.071 to 0.305)*	*85.3%* *(69.5 to 92.9%)*	*< 0.0001*	*26.5*
	*Vaccinated* vs *Unvaccinated* *(≥ 16 months-old)*	*0.100* *(0.036 to 0.273)*	*90%* *(72.7 to 86.4%)*	*< 0.0001*	*20.2*
	*Vaccinated* vs *Unvaccinated* *(≥ 20 months-old)*	*0.061* *(0.015 to 0.247)*	*93.9%* *(75.3 to 98.5%)*	*< 0.0001*	*15.3*
II	*Vaccinated* vs *Unvaccinated* *(≥ 12 months-old)*	*0.173* *(0.083 to 0.360)*	*82.7%* *(64 to 91.7%)*	*< 0.0001*	*22.1*
	Gender (Female vs Male)	1.032 (0.673 to 1.584)		0.89	0.02
	County of Residence	0.994 (0.983 to 1.005)		0.27	1.20
	*Date of Birth*	*0.999* *(0.999 to 0.999)*		*< 0.0001*	*30*
	*Vaccinated* vs *Unvaccinated* *(≥ 16 months-old)*	*0.111* *(0.040 to 0.303)*	*88.9%* *(69.7 to 96%)*	*< 0.0001*	*18.4*
	Gender (Female vs Male)	1.109 (0.718 to 1.715)		0.64	0.22
	County of Residence	0.995 (0.984 to 1.007)		0.42	0.66
	*Date of Birth*	*0.999* *(0.999 to 0.999)*		*< 0.0001*	*28.1*
	*Vaccinated* vs *Unvaccinated* *(≥ 20 months-old)*	*0.070* *(0.017 to 0.287)*	*93%* *(71.3 to 98.3%)*	*< 0.0001*	*13.7*
	Gender (Female vs Male)	1.027 (0.658 to 1.602)		0.91	0.01
	County of Residence	0.996 (0.985 to 1.008)		0.49	0.47
	*Date of Birth*	*0.999* *(0.999 to 1.000)*		*< 0.0001*	*27.4*

*Italicized* results are statistically significant. Model I = unadjusted, Model II = adjusted for gender, county of residence, and date of birth

*CI* Confidence interval, *VE* Vaccine effectiveness

^a^ Persons received only 1 dose of MMR vaccine

^b^ Persons received no doses of measles-containing vaccine

## Discussion

The results of this retrospective longitudinal cohort study of prospectively collected healthcare data provide important and compelling new epidemiological quantitative data regarding the vaccine effectiveness of the childhood MMR vaccine routinely administered to American children. Further, the vaccine effectiveness of childhood MMR vaccination remained when considering covariates such as gender, date of birth, and county of residence.

In considering the results observed in the present study with previous studies, the Cochrane Collaboration recently published an extensive review examining MMR vaccine effectiveness [7]. Of three MMR vaccine effectiveness studies examined by the Cochrane Collaboration, all three were cohort studies that observed childhood MMR vaccine administration was significantly effective in preventing clinical cases of measles [8–10]. In addition, the Cochrane Collaboration review also described that vaccine effectiveness = 97% for MMR vaccine administered in US vaccine programs [11].

The results observed in this study regarding the vaccine effectiveness of childhood MMR to significantly reduce the incidence rate of measles cases for many years post-vaccination are biologically plausible. For example, it was reported as early as 1971 that among 715 children (with no initial antibody to measles) that MMR vaccination induced a positive measles antibody response in 96% of the children [12]. It was subsequently reported that among children administered a single dose of MMR vaccine at 15 months-old and evaluated for antibodies against measles at 6–7 years-old that > 90% were still positive for antibodies against measles [13]. Other studies in different populations revealed that measles antibodies may persistent more than a decade post-MMR vaccination [14, 15]. In addition, it was even revealed in long-term longitudinal cohort studies of measles vaccine recipients that persistent antibodies were observed in > 90% of the persons examined [16, 17].

The results of the present study also revealed that vaccine effectiveness was greatest for those children administered a single dose of MMR vaccine between 16 and 20 months of age as compared to those receiving a single dose of MMR vaccine between 12 and 15 months. This type of age dependent MMR vaccine effectiveness was described in previous epidemiological studies [18]. The ACIP reported that measles vaccine is at least 95% effective for children vaccinated at ≥15 months of age, whereas a lower efficacy was observed for children vaccinated between 12 and 14 months of age. They commented that measles vaccine efficacy maybe lower because of trans-placental maternal antibody persists beyond the first birthday in some children, which, interferes with response to vaccination [19]. The biological plausibility of age dependent MMR vaccine effectiveness is supported by a previous study that revealed higher proportion of those vaccinated with MMR vaccine at younger ages had undetectable or low levels of measles antibody 5–6 years post-immunization [20]. As a consequence, there is a potential delicate balance to weigh between ensuring the best possible long-term immunological response to MMR vaccine administration in vaccine recipients versus the potential of measles exposure and measles-associated disease among children. It would seem, at least in the US, in more recent years with the end of endemic measles transmission that for most children administration of MMR vaccine at ≥15 months would be more appropriate than < 15 months, although the impact maybe limited because most children will subsequently receive a second childhood dose of MMR vaccine. It is also worth considering in countries where the risk of measles infection is high and low MMR vaccine uptake, especially for the second dose of MMR vaccine, that administration of MMR vaccine at ≥15 months maybe a means to improve long-term protection against measles infection for many children.

Another interesting aspect of the present study was that there were three measles disease diagnoses made within 30 days post-MMR vaccine (these were excluded from analyses undertaken in the present study to determine MMR vaccine effectiveness). It was previously reported the usual incubation period of measles is 8 to 12 days [21]. All three measles diagnoses appeared to occur within the biologically appropriate time for the incubation and manifestation of measles infection (one on day 11 and two on day 13), and as a result are most likely MMR vaccine-associated adverse events. It is unclear whether any previous epidemiological studies were large enough to observe this phenomenon following childhood MMR vaccine administration, but a previous day-to-day reactogenicity study of MMR vaccine versus a placebo administered to 14 to 18 months-old in a twin study revealed apparently mild measles-associated symptoms commenced 5 to 7 days post-MMR vaccine administration and peaked on day 10 [21]. The results of the present study support that MMR vaccine-associated measles adverse events are rare with a rate of 0.91 per 10,000 recipients (95% confidence interval = 0.19 to 2.67 per 10,000 recipients) within 30 days of MMR vaccination. It would be interesting in future studies to use microbiological tests to determine whether such potential measles adverse events are truly vaccine-associated or the result of wild-type measles infections.

### Strengths/limitations

An important strength of this study was that retrospective observations made in the IHRD were derived from eligibility and claims records prospectively generated as part of the routine healthcare provided for persons in the Florida Medicaid system. Therefore, the data examined were generated completely separate from the current study design. The healthcare providers submitting claims for MMR vaccine administration and measles diagnoses were most likely not thinking about the possible relationship between MMR vaccination and measles diagnoses.

The study design utilized to examine the IHRD was another important strength of the present study. All persons examined in this study were eligible for Florida Medicaid from birth for 120 months (no gaps in eligibility were allowed). In addition, in order to ensure that the cohort of persons examined was actively utilizing healthcare services from the Florida Medicaid system, all persons examined in this study had to have ≥10 outpatient office visit claims submitted (that averages to at least one outpatient office visit per person per year). These requirements helped to significantly reduce possible enrollment factors or differences in healthcare-seeking behaviors among the persons examined in this study.

Further, vaccination status was determined with precision for each person because detailed information regarding procedure codes and dates of service for claims submitted on behalf of each person were examined. In order for a person to become a member of the vaccinated cohort, the claims records for a person revealed that only a single dose of MMR vaccine was administered. Those persons with procedure codes specifying other measles-containing vaccine(s) or multiple doses of MMR vaccine were excluded from the present analyses. Similarly, members of the unvaccinated cohort were confirmed to have no claims submitted on their behalf specifying receipt of any type of measles-containing vaccine.

The outcome status was also determined with precision for each person because detailed information regarding outcomes using ICD-9 diagnosis coding and dates of service for claims submitted on behalf of each person were examined. In order for a person to be recognized as having a measles diagnosis, the initial date of service specifying a measles diagnosis (055xxx) was identified.

Finally, the use of Cox proportional hazards survival plot modeling to evaluate cases of measles diagnosed over a period of many years in the vaccinated and unvaccinated cohorts allowed for us to draw inferences regarding the relationship between vaccination and outcomes as a function of follow-up time.

It is possible that a potential limitation of this study was that the findings observed were the result of statistical chance or cofounders/unknown biases in the data. Statistical chance seems unlikely given that a limited number of statistical tests were performed, and most results were highly statistically significant. In addition, it was observed that the significant effects observed in unadjusted models remained significant even when adjusting for potential covariates such as gender, date of birth, and county of residence. The results observed this study were consistent with previous epidemiological observations on different populations and were biologically plausible.

It is also possible that some of the persons examined in the IHRD may have had symptoms of measles that were so slight that they were not noted by their healthcare providers, or healthcare providers may have misdiagnosed or misclassified vaccination status for some persons. However, these potential limitations, while possible, should not have affected the results appreciably because it is uncertain how differential application would have occurred in the vaccinated and unvaccinated cohorts examined. Importantly, any misclassification with respect to diagnostic or vaccination status, would in all likelihood bias the findings towards the null hypothesis because persons examined would have been put into the wrong vaccination and/or diagnostic category and result in diminished statistical power to establish the accurate relationship between vaccination and outcomes.

Another potential limitation of this study was that limited information was available regarding the area of residence of persons over the multiple years of this study. It was assumed that chances of exposure to wild type measles virus were equal in the vaccinated and unvaccinated cohorts examined. It is possible that there may be differences in the chances of wild type measles virus exposure in different geographical areas over different years. This potential phenomenon should be further examined in future studies.

An additional potential limitation of this study was that measles cases were not uniformly diagnosed during the study period from 1990 to 2009. As revealed in Table 5, it was observed that most cases of measles were diagnosed in the early 1990s period, and by the 2000s virtually no cases of measles were diagnosed, regardless of vaccination status. This phenomena most probably reflects increasing “herd immunity” from increasing MMR vaccine coverage in the overall population. As described previously about “herd immunity” [22], the consequence is that the chance of exposure to measles throughout the study period examined significantly decreased regardless of vaccination status, and as a result, this may have reduced the vaccine effectiveness observed in the present study in comparison with previous studies examining measles vaccine effectiveness. Namely, unvaccinated persons were deriving a benefit of protection against measles infection from vaccinated persons. It would be interesting in future studies to evaluate the impact of increasing “herd immunity” on population measles disease patterns.

**Table 5:** A summary of diagnosed measles cases by year of diagnosis

Year of Diagnosis	All Measles Cases	MMR Vaccinated Cohort^a^ Diagnosed with Measles	Unvaccinated Cohort^b^ Diagnosed with Measles
1991	18	0	18
1992	14	0	14
1993	11	1	10
1994	11	2	6
1995	8	1	7
1996	8	2	6
1997	6	1	5
1998	5	1	4
1999	4	1	3
2000	2	0	2
2001	0	0	0
2002	0	0	0
2003	1	0	1
2004	0	0	0
2005	0	0	0
2006	0	0	0
2007	0	0	0
2008	0	0	0
2009	0	0	0
Total	85	9	76

^a^Persons received only 1 dose of MMR vaccine

^b^Persons received no doses of measles-containing vaccine

A still further potential limitation of the present study was that only persons receiving a single dose of MMR vaccination were examined in the vaccinated cohort. The ACIP recommends that a second dose of MMR vaccination should be administered during childhood [1]. It is possible that additional doses of MMR vaccination may further improve vaccine effectiveness to prevent cases of measles. It is recommended that future studies further explore the impact of additional doses of MMR vaccination on vaccine effectiveness to prevent measles cases.

Another potential limitation of the present study was that the better vaccine effectiveness associated with older age of receiving MMR vaccination might associated with missing data on the first vaccination (i.e., perhaps the first documented MMR vaccination was really a second MMR vaccination, because the first was not documented). This would appear to be unlikely, since the ages examined for receipt of MMR vaccination were all before the second birthday, and the ACIP does not recommend administration of a second dose of MMR vaccine at such a young age [1].

A final potential limitation of the present study was that it is unknown how many cases were reported to the national notifiable disease system from Florida in this time period compared to what was observed in the Florida Medicaid system. It is possible that there may be some sort of selection bias, although it would probably not have a large impact on the observations made in this study.

## Conclusion

This retrospective cohort study of prospectively collected healthcare data from the IHRD provides new evidence consistent with and extending results from previous epidemiological studies revealing that routine childhood MMR vaccination in the US significantly reduced the incidence rate of diagnosed measles cases. Furthermore, MMR vaccine administration at 16–20 months-old was associated with greater vaccine effectiveness relative to MMR vaccine administration at 12–15 months-old. It was also revealed that on rare occasions in the 30-day post-MMR vaccine administration period about 1 in 10,000 doses of MMR vaccine developed MMR vaccine-associated measles adverse events. Finally, the results observed in this study help to establish that the IHRD is an important epidemiological resource to help quantitatively evaluate important public health issues.

Overall, the results of the present study support the ongoing successful use of routine MMR vaccination as an important public health tool to reduce the incidence rate of diagnosed measles. Future studies should continue to monitor how long and how robust the protective effects of MMR vaccine(s) can be expected to persist.

## Data Availability

This database is publicly available through the US Medicaid system.

## Abbreviations

AAFP: American Academy of Family Practitioners
AAP: American Academy of Pediatrics
ACIP: Advisory Committee on Immunization Practices
AHCA: Agency for Health Care Administration
CA: California
FL: Florida
ICD-9: International Code for Disease, 9th revision
IHRD: Independent Healthcare Research Database
IRB: Institutional Review Board
MMR: Measles-mumps-rubella
NDC: National Drug Codes
NJ: New Jersey
US: United States
USA: United States of America
VE: Vaccination effectiveness
WA: Washington
